# Evaluation of the real-world protective effect of mumps-containing vaccines among children under 15 years old in Taizhou: A 1:2 matched case-control study

**DOI:** 10.1371/journal.pone.0359417

**Published:** 2026-09-28

**Authors:** Huaxian Liu, Zhongkui Zhu, Yuanbao Liu, Jintao Wang, Yunke Qian, Yang Yu, Lu Shen, Yan Xie, Wanqin Tang

**Affiliations:** 1 Immunization Planning Department, Center for Disease Control and Prevention of Taizhou, Jiangsu, China; 2 Institute of Immunization Program, Center for Disease Control and Prevention of Jiangsu, Jiangsu, China; University College London, UNITED KINGDOM OF GREAT BRITAIN AND NORTHERN IRELAND

## Abstract

**Background:**

This study aimed to evaluate the real-world protective effectiveness of the mumps-containing vaccine (MuCV) among children under 15 years of age in Taizhou City, and to analyze the impact of various vaccination-related factors on vaccine effectiveness (VE), so as to provide localized epidemiological evidence for optimizing mumps immunization strategies in children.

**Methods:**

A 1:2 matched case-control study was conducted, enrolling 1,405 clinically or laboratory-confirmed mumps cases aged under 15 years in Taizhou City from 2021 to 2025, with 2,810 controls matched by sex, stage of schooling, area of residence, and date of birth. Conditional logistic regression models were used to analyze factors influencing VE. To address sparse data bias, Firth penalized logistic regression sensitivity analyses were additionally performed using R software.

**Results:**

The proportion of unvaccinated individuals was significantly higher in the case group (5.3%) than in the control group (0.5%). Receiving at least one dose of MuCV provided a VE of 75.7% (95% *CI*: 64.8% ~ 83.2%). VE showed an increasing trend with age: no significant protective effect was observed in infants and young children (aged 0 ~ 2 years); for kindergarten children (aged 3 ~ 5 years), VE was 55.2% (95% *CI*: 22.6% ~ 74.1%); for primary school children (aged 6 ~ 11 years), VE was 86.7% (95% CI: 72.8% ~ 93.5%); and for secondary school children (aged ≥12 years), VE was 92.9% (95% *CI*: 76.4% ~ 97.9%). However, sparse data bias in the younger age subgroups warrants cautious interpretation. During the single-dose immunization schedule period, VE was 80.4% (95% *CI*: 70.3% ~ 87.0%), whereas during the two-dose period, no statistically significant protective effect was observed. This result should be interpreted with caution, as the extremely small number of unvaccinated children precluded a reliable assessment of VE, and should not be interpreted as evidence that the two-dose schedule is ineffective. Using unvaccinated individuals as the reference, the *P* values for the < 18 months and ≥18 months groups were 0.456 and 0.460, respectively, with no statistical significance. Because only 14 unvaccinated controls were available, the *OR* and VE could not be reliably estimated. The interval between two doses (≤10 months vs. > 10 months) and time since last dose (≤5 years vs. > 5 years) did not show a significant effect on VE (P > 0.05). Due to the limited sample size, these results should be interpreted with caution. Sensitivity analyses and Firth penalized logistic regression analyses yielded results consistent with the main findings, collectively supporting the robustness of our conclusions.

**Conclusion:**

MuCV provided significant overall protection against mumps in children under 15 years of age. Subgroup analyses were limited by the extremely high vaccination coverage and the consequent scarcity of unvaccinated individuals. Strengthening vaccination management for infants, young children, and kindergarten children is recommended. Future studies with larger sample sizes and extended follow-up are needed to clarify the incremental value of the two-dose schedule and the pattern of waning immunity.

## Introduction

Mumps, an acute respiratory infectious disease caused by the mumps virus (MuV), is primarily transmitted via respiratory droplets or saliva, exhibiting high infectivity [1,2]. MuV infection typically presents as parotitis or swelling of other salivary glands, but it can also lead to complications including orchitis, oophoritis, hearing loss, encephalitis, and meningitis [3]. Vaccination with the mumps-containing vaccine (MuCV) is the most cost-effective and crucial measure for mumps prevention [4]. As of July 2024, 124 out of 194 (63.92%) World Health Organization member states have incorporated MuCV into their national immunization programs [5]. In most developed countries, vaccination coverage exceeds 90%, leading to a dramatic decline in mumps incidence from historically high levels of 100–1000 cases per 100,000 population to less than 1 case per 100,000 [6]. Despite this, mumps outbreaks have been reported in several countries with high vaccine coverage, including China [7], Australia [8], the United States [9], Canada [10], and Portugal [11]. Although the overall case-fatality rate of MuV infection is low, its resurgence trend globally [12] and the risk of potentially severe complications underscore that mumps remains a significant public health concern [5]. Therefore, a systematic evaluation of the real-world protective effectiveness of MuCV is of substantial public health importance for optimizing prevention and control strategies.

China introduced MuCV in the 1990s, with voluntary, self-paid vaccination for the eligible population [13]. Since 2008, MuCV has been included in the national immunization program, recommending one routine dose of the measles-mumps-rubella (MMR) vaccine for children aged 18 ~ 24 months [14]. Starting in June 2020, a two-dose MMR immunization schedule was implemented, with doses administered at 8 months and 18 months of age, respectively [15]. Surveillance data indicate that the reported mumps incidence in China decreased at an average annual rate of 6.99% between 2005 and 2023 [16]. However, despite high vaccination coverage, children under 15 years remain the primary group affected by mumps [17,18]. Furthermore, vaccine effectiveness has been suboptimal during outbreaks in congregate settings such as schools [19,20].

Since the introduction of MuCV into the national immunization program in Taizhou City in 2008, the overall incidence of mumps has shown a declining trend. Our previous research demonstrated that the average annual reported incidence during the two-dose MMR immunization strategy period (2021 ~ 2023) was 5.66 per 100,000, representing a 32.34% decrease from 8.37 per 100,000 during the single-dose strategy period (2008 ~ 2019) [18]. In Jiangsu Province, where Taizhou is located, the annual reported incidence of mumps fluctuated between 6.32 and 24.34 per 100,000 from 2004 to 2021, with an average annual incidence of 12.76 per 100,000. By 2023, the annual incidence had declined to 6.48 per 100,000. Notably, during 2020 ~ 2023, the age group with the highest mumps incidence shifted to younger ages, with an increasing proportion of cases occurring in the 0 ~ 4 year age group [21]. These epidemiological changes provided important contextual support for the subgroup analyses in the present study. Therefore, this study employed a 1:2 matched case-control design, focusing on children under 15 years in Taizhou City. The aim was to systematically evaluate the overall real-world protective effectiveness of different numbers of MuCV doses in this population. Stratified analyses were conducted across multiple dimensions, including stage of schooling, stage of immunization schedule implementation, age at first dose, time since the last dose, and interval between two doses. The study seeks to elucidate key factors influencing vaccine effectiveness, thereby providing scientific evidence and practical support for optimizing immunization strategies against mumps in children.

## Materials and methods

### Study design

This study employed a 1:2 matched case-control design to systematically evaluate the protective effectiveness of MuCV against mumps. The study subjects were all from Taizhou City, Jiangsu Province, China. Cases were sourced from the mumps surveillance database of the China Disease Prevention and Control Information System. The mumps surveillance database included all clinically diagnosed and laboratory-confirmed cases reported by medical institutions at all levels in Taizhou City, based on the national health industry standard “Diagnostic Criteria for Mumps” (WS 270–2007) [22]. Case Inclusion Criteria: The case group comprised clinically diagnosed or laboratory-confirmed mumps cases meeting the following criteria: aged 0 ~ 14 years; year of onset between 2021 and 2025; permanent resident of Taizhou City; and willing to participate in the survey. The period 2021 ~ 2025 was selected as the study period for the following reasons: (1) it spanned the transition from the single-dose to the two-dose immunization schedule (the two-dose schedule was implemented in June 2020), allowing for comparison of vaccine effectiveness (VE) between the two immunization strategy periods; and (2) the study was initiated in 2025, and all cases from the most recent complete years prior to that were included, during which data completeness and quality were at their highest levels.

Control Matching Criteria: Controls were matched to cases at a 1:2 ratio based on the following criteria: same sex; same stage of schooling; same community/village (if a match was not available within the same community/village, a control was selected from a neighboring community/village); date of birth within ±1 month; willing to participate and not belonging to the same household as the matched case; confirmed via household visit or telephone follow-up to have no history of symptoms related to the parotid or other salivary glands; and no record of clinical or laboratory-diagnosed mumps in the China Disease Prevention and Control Information System.

The survey was conducted from February 2025 to February 2026. From an initial pool of 1498 eligible cases, 1405 cases (response rate 93.8%) completed the survey and were included in the final analysis, with 93 cases lost to follow-up (loss-to-follow-up rate 6.2%). A total of 2810 controls were selected according to the 1:2 matched design. All subjects underwent rigorous screening and data verification to ensure data completeness and comparability between groups.

MuCV vaccination histories for both cases and controls were primarily retrieved from the Jiangsu Provincial Immunization Information System. For individuals with incomplete or missing records in the system, information was supplemented and verified via household inspection of immunization certificates or telephone inquiries. Collected information included vaccination status (yes/no), vaccine name, vaccination date, and number of doses. Vaccine types included monovalent mumps vaccine (MuV), measles-mumps combined vaccine (MM), and measles-mumps-rubella combined vaccine (MMR).

A breakthrough case was defined as a mumps case occurring more than 42 days (1.5 times the maximum incubation period) after MuCV vaccination [23]. Accordingly, a MuCV dose administered >42 days before the date of disease onset was considered a valid immunization record, indicating that the child had sufficient time to mount an immune response prior to exposure. A dose administered ≤42 days before onset or after onset was not considered protective due to the timing of exposure. For controls, vaccination status was assessed using the onset date of the matched case as the reference time point (i.e., in each matched set, the vaccination histories of the case and its two controls were all evaluated relative to the case’s onset date). This design ensured that all participants within each matched set shared the same observation window, thereby maintaining temporal comparability required for the case-control study. According to their MuCV vaccination history, subjects were categorized into 0-dose, 1-dose, and 2-dose groups (Fig 1.).

**Fig 1:**
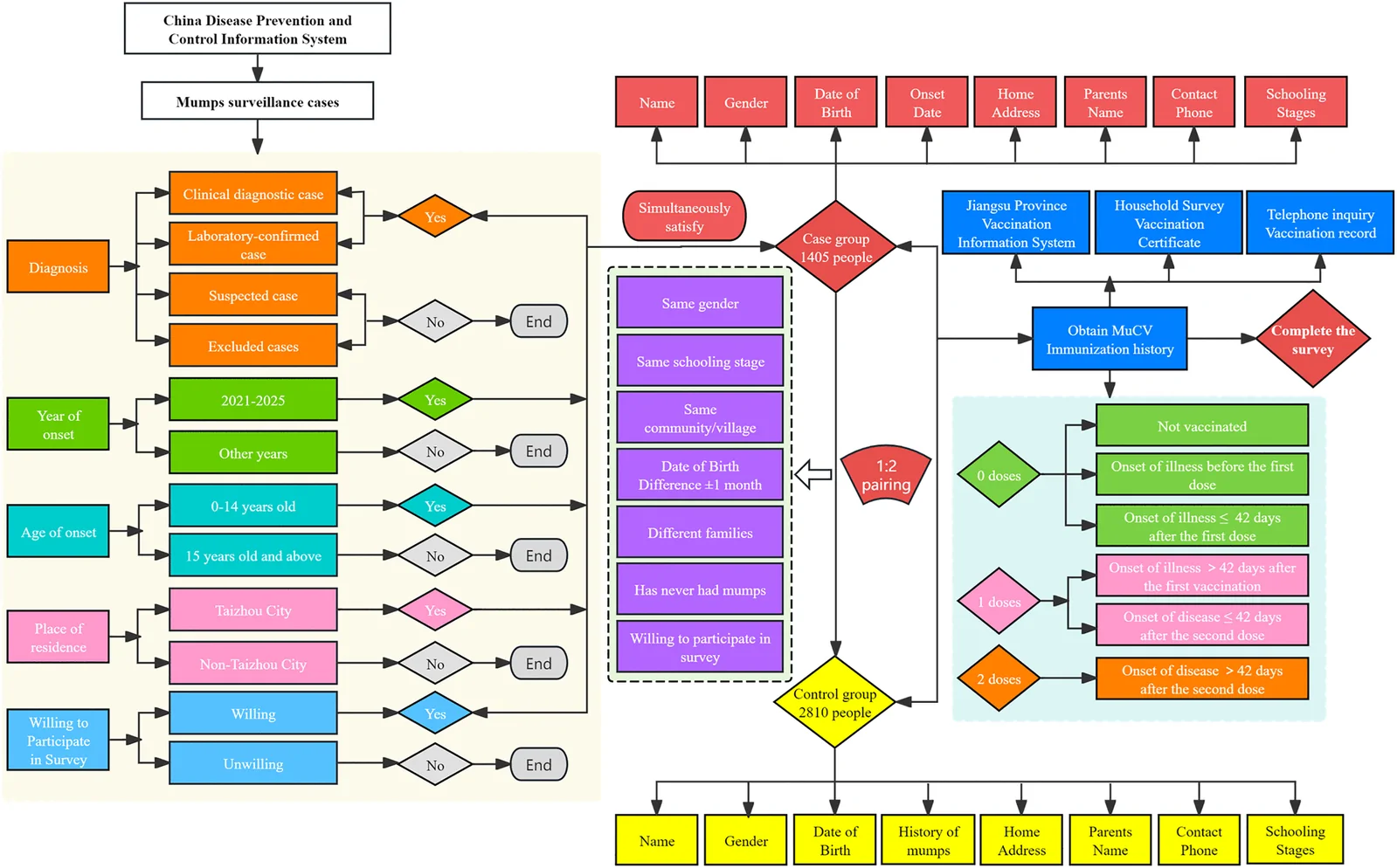
Flowchart of the 1:2 matched case-control study on MuCV vaccine effectiveness among children under 15 years of age in Taizhou.

### Sample size

This study employed a 1:2 matched case-control design. The sample size was calculated based on the formula for matched case-control studies [24].

$n={\left[Z_{\alpha }\sqrt{(\text{1}+\text{1}/r)\overset{―}{P}\left(\text{1}-\overset{―}{P}\right)}+Z_{\beta }\sqrt{P_{\text{1}}\left(\text{1}-P_{\text{1}}\right)/r+P_{\text{0}}\left(\text{1}-P_{\text{0}}\right)}\right]}^{\text{2}}/{\left(P_{\text{1}}-P_{\text{0}}\right)}^{\text{2}}$


$$\begin{array}{c} P_{\text{1}}=\left(OR\times P_{\text{0}}\right)/\left(\text{1}-P_{\text{0}}+OR\times P_{\text{0}}\right) \end{array}$$

$$\begin{array}{c} \overset{―}{P}=\left(P_{\text{1}}+rP_{\text{0}}\right)/(\text{1}+r) \end{array}$$

The parameters were set as follows: Type I error probability α = 0.05 (Zα = 1.96), Type II error probability *β* = 0.10 (Z_*β*_ = 1.28), number of matched controls per case r = 2. Based on the surveillance data of MuCV vaccination coverage among children under 15 years of age in Taizhou City from 2021 to 2025, the vaccination proportion in the control group ($P_{\text{0}}$) was set at 0.99. Referring to previous research, the expected MuCV protective effectiveness was 70% [25], corresponding to an odds ratio (*OR*) of 0.3. Substituting these values into the formula yielded a required sample size of 1,108 cases and 2,216 controls.

To account for potential loss to follow-up, the calculated sample size was increased by 20%, resulting in a final minimum requirement of at least 1,330 cases and 2,660 controls. According to the mumps surveillance data from the China Information System for Disease Control and Prevention, a total of 1,405 clinically diagnosed or laboratory-confirmed mumps cases aged 0 ~ 14 years in Taizhou City from 2021 to 2025 were eligible and willing to participate in the survey. The number of available cases (1,405) essentially met the above minimum sample size requirement (1,330), providing the necessary statistical basis for the primary analyses and major subgroup analyses in this study. For subgroups with relatively small sample sizes, the results are presented as exploratory analyses and should be interpreted with caution.

### Survey content

This study investigated the protective effectiveness of MuCV among children under 15 years in Taizhou City. The survey content primarily comprised two main categories: basic information and MuCV vaccination history (Table 1). Basic information included the child’s name, sex, date of birth, home address, history of mumps, stage of schooling, as well as parents’ names and contact numbers. The MuCV vaccination history focused on collecting the child’s vaccination records, including key indicators such as vaccine name, vaccination date/time, and number of doses. To ensure the accuracy and completeness of vaccination data, all vaccine-related information was verified and cross-checked against the Jiangsu Provincial Immunization Program Information Management System.

**Table 1:** Survey content of MuCV protection effect study in children under 15 years old in Taizhou City.

Survey category	Survey content
Basic Information	Name
	Gender
	Date of Birth
	Home Address
	History of Mumps
	Stage of Schooling
	Father’s Name
	Mother’s Name
	Contact Phone
MuCV Vaccination	Vaccination History
	Vaccine Name
	Vaccination Time
	Number of Doses

Regarding data collection and management, after paper questionnaires were collected, dedicated personnel uniformly entered the data into the electronic platform “Wenjuanxing” (Questionnaire Star), followed by a dedicated review process. Replacing traditional paper questionnaires with electronic data collection significantly improved survey efficiency. This platform supports various question types and logic skip functions, allowing for flexible adaptation to complex survey needs. Furthermore, data validation measures such as mandatory question settings and format restrictions significantly reduced data entry errors caused by non-standardized responses and minimized omissions and biases during data transcription.

### Quality control

Prior to the formal survey, the Taizhou Center for Disease Control and Prevention (Taizhou CDC) developed a detailed survey protocol and conducted systematic training for professional technical staff from county/district-level CDCs. After data collection, investigators reviewed the completeness of each individual questionnaire. Questionnaires were signed and confirmed only after verification that there were no missing or omitted items. Quality control personnel audited the questionnaires, and if issues were identified, investigators were required to make corrections through re-investigation or supplementary inquiry. Following data collection, county/district-level CDCs were responsible for collating and entering data from their respective jurisdictions. The Taizhou CDC then integrated the city-wide database and performed logical consistency checks on the data. Furthermore, designated personnel cross-referenced the basic information and MuCV vaccination history of the study subjects against the Jiangsu Provincial Immunization Program Information Management System to further ensure data accuracy and reliability.

Additionally, to enhance the scientific rigor and feasibility of the individual questionnaires, a pilot survey involving 30 parents of children was conducted before the formal study. The questionnaire was optimized and adjusted based on issues identified during this pilot.

### Statistical analysis

Data exported from the “Questionnaire Star” platform were organized into Excel format. Statistical analysis was performed using SPSS software (version 25.0). Categorical variables were compared between groups using the *χ²* test. Given the 1:2 matched case-control design, conditional logistic regression models were applied to analyze the odds ratios (*OR*) and their 95% confidence intervals (*CI*s) for associations between case/control status and various factors. These factors included the number of vaccine doses received, stage of schooling, stage of immunization schedule implementation, age at first dose, time since the last dose, and interval between two doses. The *OR* was used to estimate the relative risk (*RR*). Vaccine effectiveness (VE) was calculated using the formula: VE=(1−RR)×100% [26], where *OR* is the odds ratio derived from the conditional logistic regression model, representing the odds of mumps occurrence among vaccinated individuals relative to unvaccinated individuals. This formula estimates the proportion of disease risk reduced by MuCV vaccination.The significance level (*α*) was set at 0.05.

To address potential sparse data bias due to the extremely small number of unvaccinated individuals in certain subgroups, we further performed Firth penalized logistic regression sensitivity analyses using R software (version 4.6.0, logistf package), with the results serving as complementary validation of the primary findings. In addition, to evaluate whether the vaccination effect varied across schooling stages and to obtain estimates that borrow information across strata, we fitted a single conditional logistic regression model (stratified by matched pair identifier) incorporating a dose-by-schooling-stage interaction term across the full sample. The statistical significance of the interaction term was assessed using the likelihood ratio test. Furthermore, to verify the rationality of the cutoff values used in the categorical variable analyses, we conducted sensitivity analyses by entering age at first dose, time since last dose, and interval between two doses as continuous variables into conditional logistic regression models, respectively, to assess whether linear trends existed between these factors and the risk of mumps.

### Ethical review and informed consent

This study was reviewed and approved by the Ethics Committee of Taizhou Hospital of Integrated Traditional Chinese and Western Medicine (Approval No.: 2023-Ethics Review LW-001, Approval Date: May 22, 2023). The entire research process strictly adhered to ethical standards. All surveys were conducted only after obtaining written informed consent signed by the parents or legal guardians of the study subjects. The research purpose, procedures, and scope of data use were explained in detail.

During the implementation of the study, strict confidentiality protocols were followed. All data were stored and analyzed in anonymized form, used exclusively for this research, and were not disclosed to any third party without authorization. The research team was fully committed to protecting the personal privacy and data security of all participants.

## Results

### Characteristics of the study subjects

A total of 1405 cases meeting all inclusion criteria completed the survey. Using a 1:2 matched design, 2810 controls were included. The distribution of sex, stage of schooling, residence, and birth period (before or after October 1, 2019) was identical between the case and control groups, indicating good comparability. Among the 1405 cases, 1108(78.9%) were clinically diagnosed and 297(21.1%) were laboratory-confirmed. During the study period, MuCV vaccination coverage among control children showed an upward trend, increasing from 97.2% in 2021 to 99.8% in 2025, reflecting the continued implementation of the immunization program. Regarding sex distribution, males accounted for 62.9% of the case group, compared to 37.1% for females. In terms of stage of schooling, primary school children constituted the largest proportion (39.6%), followed by kindergarten children (29.2%). Geographically, the subjects covered six districts/counties of Taizhou City, with Jiangyan District (37.4%) and Taixing City (29.4%) being the primary areas. Regarding birth period, individuals born before October 1, 2019, accounted for 88.4% of the study population (Table 2).

**Table 2:** Distribution of basic characteristics between case and control groups in 1405 mumps cases in Taizhou City.

Variable	Grouping	Cases (Composition Ratio %)	Controls (Composition Ratio %)
Gender	Male	884(62.9)	1768(62.9)
	Female	521(37.1)	1042(37.1)
Schooling Stage	Infants and young children	65(4.6)	130(4.6)
	Kindergarten	410(29.2)	820(29.2)
	Primary School	557(39.6)	1114(39.6)
	Middle School	373(26.5)	746(26.5)
Residence	Hailing District	201(14.3)	402(14.3)
	Gaoxin District	76(5.4)	152(5.4)
	Jiangyan District	526(37.4)	1052(37.4)
	Xinghua city	39(2.8)	78(2.8)
	Jingjiang City	150(10.7)	300(10.7)
	Taixing City	413(29.4)	826(29.4)
Born Before October 1, 2019	No	163(11.6)	326(11.6)
	Yes	1242(88.4)	2484(88.4)

Note: As a 1:2 matched design was adopted, the distributions of matching variables (gender, stage of schooling, residence, and birth period) were completely consistent between the case and control groups; therefore, no statistical comparison between groups was performed.

### Overall protective effectiveness of MuCV by number of doses

Vaccination status (≥1 dose vs. unvaccinated) was entered into a conditional logistic regression model (case group = 1, control group = 0), with unvaccinated individuals as the reference. The results showed that receiving ≥1 dose of MuCV was a protective factor (*B*=−1.415, *P* < 0.01), with an *OR* of 0.243 (95% *CI*: 0.168 ~ 0.352), yielding a VE of 75.7% (95% *CI*: 64.8% ~ 83.2%). Regarding vaccination distribution, the proportion of unvaccinated individuals was 5.3% in the case group versus 0.5% in the control group; the proportions of those receiving at least one dose were 94.7% and 99.5%, respectively (Table 3).

**Table 3:** Overall protective effectiveness of MuCV by number of doses.

Variable	Number of Cases (Composition Ratio)	Number of Controls (Composition Ratio)	*B* Value	*Wald* *χ2* Value	*P* Value	*OR* Value (95% *CI*)	VE(%) (95% *CI*)
Dose of Vaccination			−1.415	55.758	**<0.01** ^*^	0.243 (0.168 ~ 0.352)	75.7 (64.8 ~ 83.2)
Unvaccinated (Reference)	75(5.3)	14(0.5)					
≥1 Dose	1330(94.7)	2796(99.5)					

Note: *B* is the regression coefficient from the conditional logistic regression model.

### Protective effectiveness of MuCV among children in different stages of schooling

The association between stage of schooling and protection was analyzed using conditional logistic regression (case group = 1, control group = 0), with unvaccinated children in each stage of schooling as the reference. The results showed that for Infants and young children (0 ~ 2 years), vaccination did not demonstrate a statistically significant protective effect (*P* = 0.656). Among kindergarten children (3 ~ 5 years), vaccination showed a significant protective effect (*OR* = 0.448, 95% *CI*: 0.259 ~ 0.774, *P* < 0.01), with a VE of 55.2% (95% *CI*: 22.6% ~ 74.1%). In primary school children (6–11 years), the vaccine’s protective effect was more pronounced (*OR* = 0.133, 95% *CI*: 0.065 ~ 0.272, *P* < 0.01), yielding a VE of 86.7% (95% *CI*: 72.8% ~ 93.5%). Among secondary school children (≥12 years), the protective effect was highest (*OR* = 0.071, 95% *CI*: 0.021 ~ 0.236, *P* < 0.01), corresponding to a VE of 92.9% (95% *CI*: 76.4% ~ 97.9%). However, the kindergarten and primary school control groups had zero unvaccinated individuals, and the middle school control group had only 2 unvaccinated individuals (0.3%), all of which posed a risk of sparse data bias; therefore, the results should be interpreted with caution.

To evaluate the impact of sparse data on the results, we further performed Firth penalized logistic regression sensitivity analyses. The corrected estimates for each stratum were generally consistent with the main analysis (Table 4), and the conclusions remained unchanged. Due to the sparsity of data in these schooling strata, the relevant findings should be considered exploratory.

**Table 4:** MuCV protection effect by different schooling stages.

Variable	Number of Cases (Composition Ratio)	Number of Controls (Composition Ratio)	*B* Value	*Wald* *χ2* Value	*P* Value	*OR* Value*(95%CI*)	VE(%)(95% *CI*)	Firth correction of *OR* Value(95% *CI*)	Firth correction of VE(%)(95% *CI*)
Infants and young children (0 ~ 2 years)			0.305	0.199	0.656	1.356(0.356 ~ 5.171)		1.308 (0.361 ~ 4.735)	
Unvaccinated (Reference)	8(12.3)	12(9.2)							
1 Dose	15(23.1)	40(30.8)							
2 Doses	42(64.6)	78(61.0)							
Kindergarten (3 ~ 5 years)			−0.804	8.269	**<0.01** ^*^	0.448(0.259 ~ 0.774)	55.2(22.6 ~ 74.1)	0.453 (0.263 ~ 0.780)	54.7 (22.0 ~ 73.7)
Unvaccinated (Reference)	11(2.7)	0(0.0)							
1 Dose	273(66.6)	556(67.8)							
2 Doses	126(30.7)	264(32.2)							
Primary School (6 ~ 11 years)			−2.017	30.438	**<0.01** ^*^	0.133(0.065 ~ 0.272)	86.7(72.8 ~ 93.5)	0.139 (0.069 ~ 0.280)	86.1 (72.0 ~ 93.1)
Unvaccinated (Reference)	33(5.9)	0(0.0)							
1 Dose	512(91.9)	1081(97.0)							
2 Doses	12(2.2)	33(3.0)							
Middle School (≥12 years)			−2.649	18.611	**<0.01** ^*^	0.071(0.021 ~ 0.236)	92.9(76.4 ~ 97.9)	0.081 (0.026 ~ 0.249)	91.9 (75.1 ~ 97.4)
Unvaccinated (Reference)	23(6.2)	2(0.3)							
1 Dose	347(93.0)	739(99.0)							
2 Doses	3(0.8)	5(0.7)							

Note: *B* is the regression coefficient from the conditional logistic regression model.

### Protective effectiveness of MuCV in different immunization schedule periods

The association between the period of immunization schedule implementation and protection was analyzed using conditional logistic regression (case group = 1, control group = 0), with unvaccinated individuals in the single-dose immunization schedule period and the two-dose immunization schedule period as the reference, respectively. The results revealed a significant difference in MuCV protective effectiveness between the two immunization schedule periods. During the period of the single-dose immunization schedule, vaccination demonstrated a significant protective effect (*OR* = 0.196, 95% *CI*: 0.130 ~ 0.297, *P* < 0.01), with a VE of 80.4% (95% *CI*: 70.3% ~ 87.0%). Regarding the distribution of vaccination status in this period, the proportion of unvaccinated individuals in the case group (5.5%) was significantly higher than in the control group (0.1%). The proportions of those who received 1 dose were 91.5% (cases) and 96.2% (controls), respectively. The proportions of those who received 2 doses were relatively low in both groups, at 3.0% and 3.7%, respectively. During the two-dose immunization schedule period, the case group comprised only 163 cases and the control group 326 controls, a sample size considerably smaller than that of the single-dose period (1,242 cases and 2,486 controls). Vaccination during this period did not demonstrate a statistically significant protective effect (*OR* = 1.245, 95% *CI*: 0.398 ~ 3.892, *P* = 0.706). The vaccination distributions in the case and control groups during this period were similar: the proportions of unvaccinated individuals were 4.3% and 3.7%, respectively; the proportions receiving 1 dose were 6.1% and 8.0%, respectively; and the proportions receiving 2 doses were 89.6% and 88.3%, respectively (Table 5). Given the small sample size in the two-dose period, the statistical power was limited, and the results should be interpreted with caution.

**Table 5:** MuCV protective effectiveness by immunization schedule period.

Variable	Number of Cases (Composition Ratio)	Number of Controls (Composition Ratio)	*B* Value	*Wald* *χ2* Value	*P* Value	*OR*Value (95% *CI*)	VE(%) (95% *CI*)
Single – dose Immunization Schedule Period			−1.628	59.444	**<0.01** ^*^	0.196 (0.130 ~ 0.297)	80.4 (70.3 ~ 87.0)
Unvaccinated (Reference)	68(5.5)	2(0.1)					
1 Dose	1137(91.5)	2390(96.2)					
2 Doses	37(3.0)	92(3.7)					
Two – dose Immunization Schedule Period			0.219	0.142	0.706	1.245 (0.398 ~ 3.892)	
Unvaccinated (Reference)	7(4.3)	12(3.7)					
1 Dose	10(6.1)	26(8.0)					
2 Doses	146(89.6)	288(88.3)					

Note: *B* is the regression coefficient from the conditional logistic regression model. The analyses for the single-dose and two-dose periods were conducted independently, with unvaccinated individuals within each period serving as the reference.

### Protective effectiveness of MuCV by age at first dose

Among the 1405 case-control sets, the proportion of individuals whose age at first dose was ≥ 18 months was lower in the case group (81.0%) compared to the control group (85.4%). The difference in the distribution of age at first dose between the two groups was statistically significant (*χ²* = 106.221, *P* < 0.01). To directly evaluate the impact of age at first dose on vaccine effectiveness, we used unvaccinated individuals as the reference and categorized vaccinated participants into two groups according to age at first dose: < 18 months and ≥18 months. Conditional logistic regression stratified by matched pair identifier was performed. The results showed that the *P* values for the < 18 months and ≥18 months groups were 0.456 and 0.460, respectively, with no statistical significance. Given that only 14 unvaccinated controls were available, the *OR* and VE estimates were extremely unstable, indicating complete separation and sparse data bias; therefore, the protective effect could not be reliably estimated, and *OR* and VE were not reported (Table 6).

**Table 6:** Protective effectiveness of the MuCV by age at first dose.

Age at first dose	Number of Cases (Composition Ratio)	Number of Controls (Composition Ratio)	*B* Value	*Wald* *χ2* Value	*P* Value
Unvaccinated(Reference)	75(5.3)	14(0.5)			
<18 months	192(13.7)	395(14.1)	−10.238	0.556	0.456
≥18 months	1138(81.0)	2401(85.4)	−10.147	0.547	0.460

Note: *B* is the regression coefficient from the conditional logistic regression model.

It is worth noting that age at first dose is closely related to the immunization schedule period, as the recommended age for the first dose was 18 months during the single-dose period and 8 months during the two-dose period. Consequently, it may be difficult to completely disentangle the independent effects of age at first dose from those of the immunization schedule period on VE.

### Protective effectiveness of MuCV by time since last dose

In the 1,405 matched case-control pairs, the distribution of time since last dose differed significantly between the case and control groups (*χ²* = 106.411, *P* < 0.01). Among unvaccinated individuals, the proportion was 5.3% in the case group, significantly higher than the 0.5% in the control group. Among vaccinated individuals, the proportion with time since last dose ≤5 years was 48.0% in the case group and 49.6% in the control group; the proportion with time since last dose >5 years was 46.7% in the case group and 49.9% in the control group. The distributions of vaccination intervals were similar between the two groups among vaccinated individuals, but the proportion of unvaccinated individuals was markedly higher in the case group (Table 7).

**Table 7:** Distribution of time since last dose among 1405 case-control pairs in Taizhou city.

Variable	Grouping	Number of Cases (Composition Ratio)	Number of Controls (Composition Ratio)	*χ* *2 Value*	*P* Value
Time since last dose(years)	Unvaccinated	75(5.3)	14(0.5)	106.411	**<0.01** ^*^
	≤ 5 years	674(48.0)	1393(49.6)		
	> 5 years	656(46.7)	1403(49.9)		

Among participants in whom both the case and their matched controls had received at least one dose of MuCV, the effect of time since last dose on vaccine effectiveness was evaluated using conditional logistic regression stratified by matched pair identifier (case group = 1, control group = 0), with the shortest time since last dose interval as the reference within each analysis. In the one-dose recipients, > 5 years compared with ≤5 years showed no statistical significance (*OR* = 1.164, 95% *CI*: 0.500 ~ 2.709, *P* = 0.725); similar results were observed in the ≥ 1-dose recipients (*OR* = 1.239, 95% *CI*: 0.607 ~ 2.531, *P* = 0.556). Further stratification of time since last dose into ≤2 years, 2 ~ 5 years, 5 ~ 10 years, and >10 years among one-dose recipients showed that only the > 10 years group reached statistical significance (*OR* = 6.020, 95% *CI*: 1.010 ~ 35.865, *P* = 0.049), suggesting that detectable waning of protection following a single dose of MuCV may become apparent only after 10 years, whereas no statistically significant differences were observed for shorter intervals. Due to the limited sample size, these findings should be interpreted with caution (Table 8).

**Table 8:** MuCV protective effectiveness by time since last dose.

Analyze the population	Time since last dose	Number of Cases (Composition Ratio)	Number of Controls (Composition Ratio)	*B* Value	*Wald* *χ2* Value	*P* Value	*OR* Value*(95%* *CI*)
1-dose recipients	≤5 years(Reference)	495(43.2)	985(43.1)				
	>5 years	652(56.8)	1299(56.9)	0.152	0.124	0.725	1.164(0.500 ~ 2.709)
≥1-dose recipients	≤5 years(Reference)	674(50.7)	1353(50.9)				
	>5 years	656(49.3)	1307(49.1)	0.214	0.346	0.556	1.239(0.607 ~ 2.531)
1-dose recipients	≤2 years(Reference)	48(4.2)	116(5.1)				
	2 ~ 5 years	339(29.6)	652(28.5)	1.259	3.579	0.059	3.520(0.956 ~ 12.966)
	5 ~ 10 years	402(35.0)	803(35.2)	1.448	3.768	0.052	4.257(0.986 ~ 18.376)
	>10 years	358(31.2)	713(31.2)	1.795	3.886	0.049	6.020(1.010 ~ 35.865)

Note: *B* is the regression coefficient from the conditional logistic regression model.

### Protective effectiveness of MuCV by interval between two doses

Among the population that received 2 doses of MuCV, the effect of the interval between the two doses was analyzed using conditional logistic regression (case group = 1, control group = 0), with those having an interval of ≤10 months as the reference group. The results indicated that the interval between the two doses (*OR*= 1.058, 95% *CI*: 0.599 ~ 1.871, *P* = 0.845) did not demonstrate a statistically significant effect on MuCV vaccine effectiveness. The sample size was limited, and the results should therefore be interpreted with caution. Regarding the distribution, the proportion of individuals with an interval >10 months was 16.9% in the case group and 16.5% in the control group, indicating a largely similar composition between the two groups (Table 9).

**Table 9:** MuCV vaccine protection effect by different intervals between two doses.

Variable	Number of Cases (Composition Ratio)	Number of Controls (Composition Ratio)	*B* Value	*Wald* *χ2* Value	*P* Value	*OR* Value (95% *CI*)
Interval between two doses			0.057	0.038	0.845	1.058 (0.599 ~ 1.871)
≤10 months (Reference)	152(83.1)	314(83.5)				
>10 months	31(16.9)	62(16.5)				

Note: *B* is the regression coefficient from the conditional logistic regression model.

### Sensitivity analyses

#### Firth penalized logistic regression.

To address the risk of sparse data bias in certain subgroups, we further performed Firth penalized logistic regression sensitivity analyses using R software. The results showed that the effect estimates for all variables were generally consistent with the main analysis, with none reaching statistical significance (*P* > 0.05), suggesting that the study conclusions were not affected by sparse data bias (Table 10).

**Table 10:** Sensitivity analysis using Firth penalized logistic regression.

Variable	Analyze the crowd	*B* Value	*SE*	*Z* Value	*P* Value	*OR* Value (95% *CI*)
Age at first dose	≥1 Dose	−0.005	0.007	−0.713	0.476	0.995(0.981 ~ 1.009)
Time since last dose (years)	≥1 Dose	0.003	0.010	0.324	0.746	1.003(0.984 ~ 1.023)
Interval between two doses (months)	≥2 Doses	−0.003	0.014	−0.233	0.816	0.997(0.970 ~ 1.025)

Note: *B* is the regression coefficient from the Firth penalized logistic regression model.

### Continuous variable sensitivity analysis

To verify the rationality of the cutoff values used in the categorical variable analyses, we entered age at first dose, time since last dose, and interval between two doses as continuous variables into conditional logistic regression models, respectively. The results showed that age at first dose (*OR* = 0.991, 95% *CI*: 0.972 ~ 1.010, *P* = 0.344), time since last dose (*OR* = 1.128, 95% *CI*: 0.958 ~ 1.327, *P* = 0.148), and interval between two doses (*OR* = 1.072, 95% *CI*: 0.966 ~ 1.189, *P* = 0.191) were not significantly linearly associated with the risk of mumps, suggesting that these factors did not show a significant linear effect on MuCV vaccine effectiveness, consistent with the subgroup analysis findings (Table 11). Due to the limited sample size in the analysis populations (particularly for the interval between two doses, which was restricted to those who received ≥2 doses), the results should be interpreted with caution.

**Table 11:** Sensitivity analysis results for continuous variables.

Variable	Analyze the crowd	*B* Value	*Wald* *χ2* Value	*P* Value	*OR* Value (95% *CI*)
Age at first dose	≥1 Dose	−0.009	0.896	0.344	0.991(0.972 ~ 1.010)
Time since last dose (years)	≥1 Dose	0.120	2.091	0.148	1.128(0.958 ~ 1.327)
Interval between two doses (months)	≥2 Doses	0.069	1.712	0.191	1.072(0.966 ~ 1.189)

Note: *B* is the regression coefficient from the conditional logistic regression model.

### Dose-by-schooling-stage interaction model

To further verify the statistical significance of VE differences across schooling stages, we fitted a conditional logistic regression model incorporating a dose-by-schooling-stage interaction term in the full sample, with schooling stage treated as an ordinal variable (1 = infants and young children, 2 = kindergarten, 3 = primary school, 4 = middle School). The interaction effect was statistically significant (*χ²* = 19.06, *P* < 0.001). Marginal effect estimates derived from this model showed that VE estimates for each schooling stage were highly consistent with those from the stratified analyses and Firth-corrected estimates in Table 4, indicating that the protective effect of vaccination against mumps risk increased significantly with higher schooling stages, further supporting the finding of increasing VE with age in Table 4. Moreover, due to borrowing information across strata, the confidence intervals from the interaction model were narrower than those from the stratified analyses, further confirming that the main estimates were not substantially affected by sparse data bias (Table 12).

**Table 12:** Marginal effect analysis of the interaction term between vaccination dose and schooling stages.

Schooling stages	Interaction model *OR* (95% *CI*)	Interaction model VE (%) (95% *CI*)
Infants and young children (0 ~ 2 years)	1.247(0.550 ~ 2.829)	
Kindergarten (3 ~ 5 years)	0.438(0.281 ~ 0.681)	56.2(31.9 ~ 71.9)
Primary School (6 ~ 11 years)	0.153(0.094 ~ 0.251)	84.7(74.9 ~ 90.6)
Middle School (≥12 years)	0.054(0.022 ~ 0.133)	94.6(86.7 ~ 97.8)

### Distribution characteristics of continuous variables

To illustrate the rationale for selecting the cutoff values for categorical variables, we plotted distribution histograms for age at first dose, time since last dose, and interval between two doses. The results showed that age at first dose was concentrated around 18 months in the study population (Fig 2). Time since last dose was mainly distributed between 2 and 5 years; although some local fluctuations were observed beyond 5 years, the sample size in this region was limited (only 4 cases and 8 controls receiving two doses in the > 5 years group), and the distribution was unstable. Therefore, we used 5 years as the cutoff for subgroup analysis (Fig 3). The interval between two doses was mostly distributed within the 6 ~ 14 months range, with 10 months representing the median of this distribution (Fig 4).

**Fig 2:**
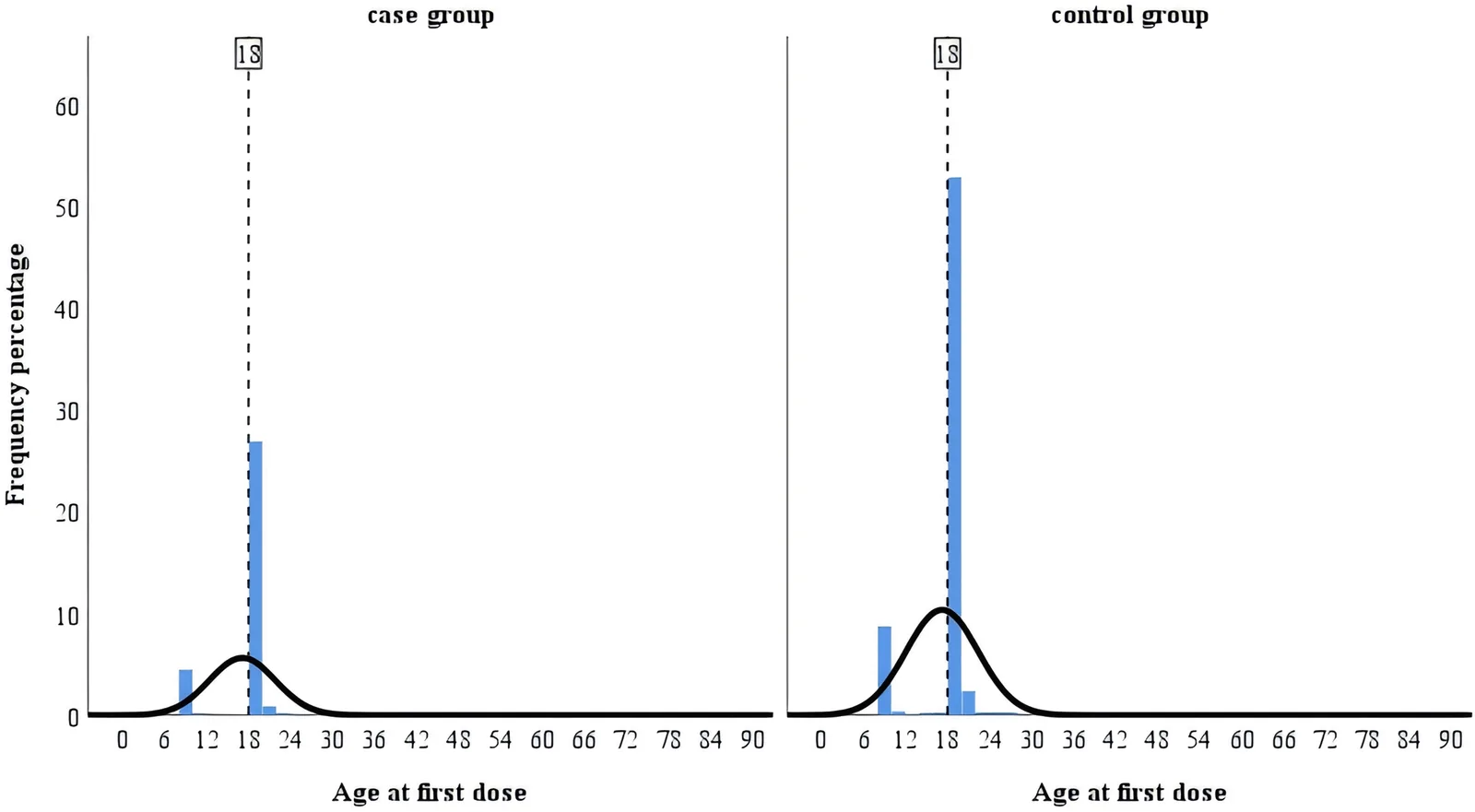
Distribution of age at first dose (months). Note: The left panel shows the case group and the right panel shows the control group. The dashed line indicates the cutoff (18 months). Both groups showed a concentration around 18 months, supporting the use of 18 months as the cutoff.

**Fig 3:**
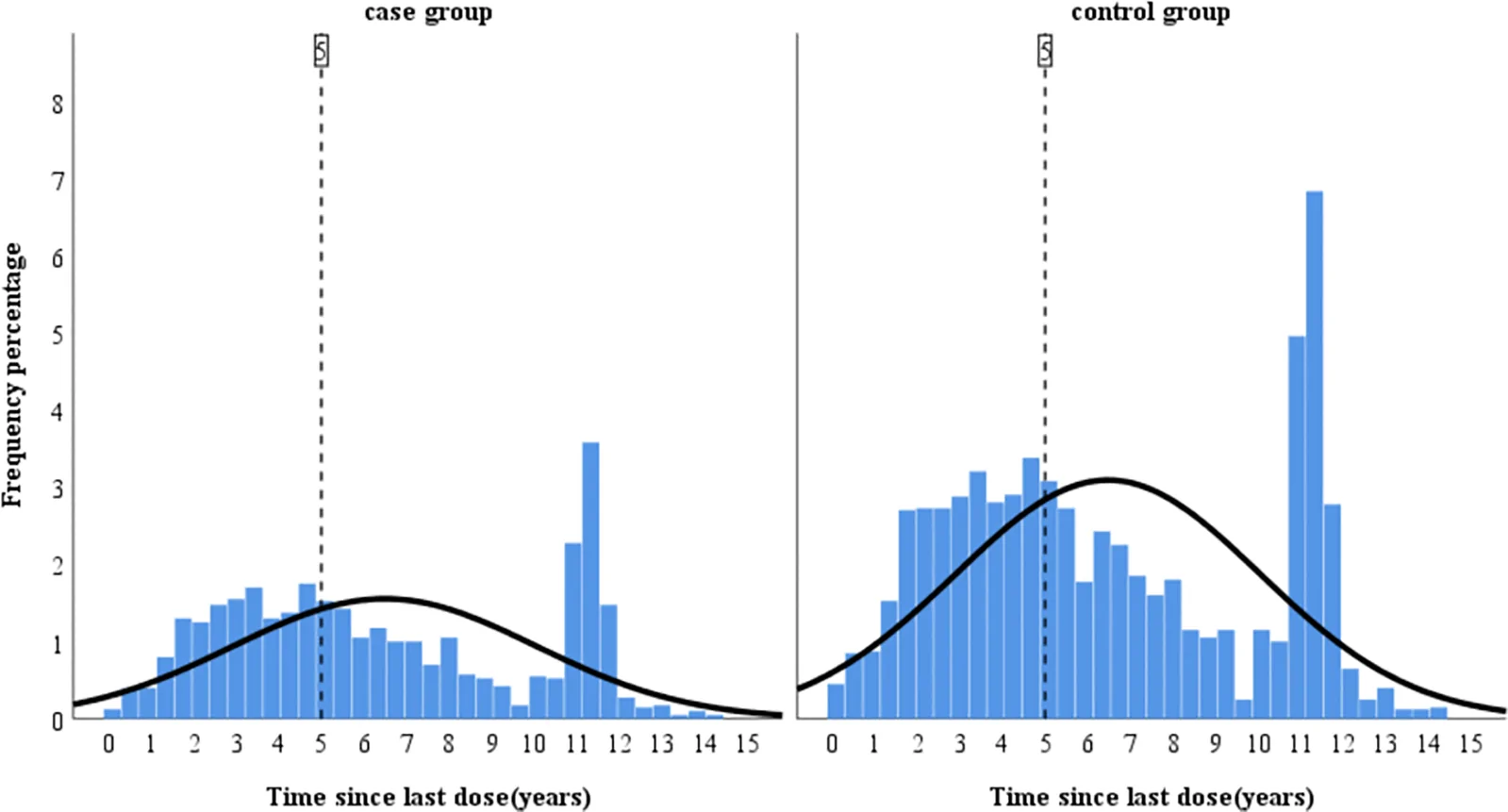
Distribution of time since last dose (years). Note: The left panel shows the case group and the right panel shows the control group. The dashed line indicates the cutoff (5 years). Due to the limited sample size in the > 5 years region, the distribution showed fluctuations and should be interpreted with attention to the overall trend.

**Fig 4:**
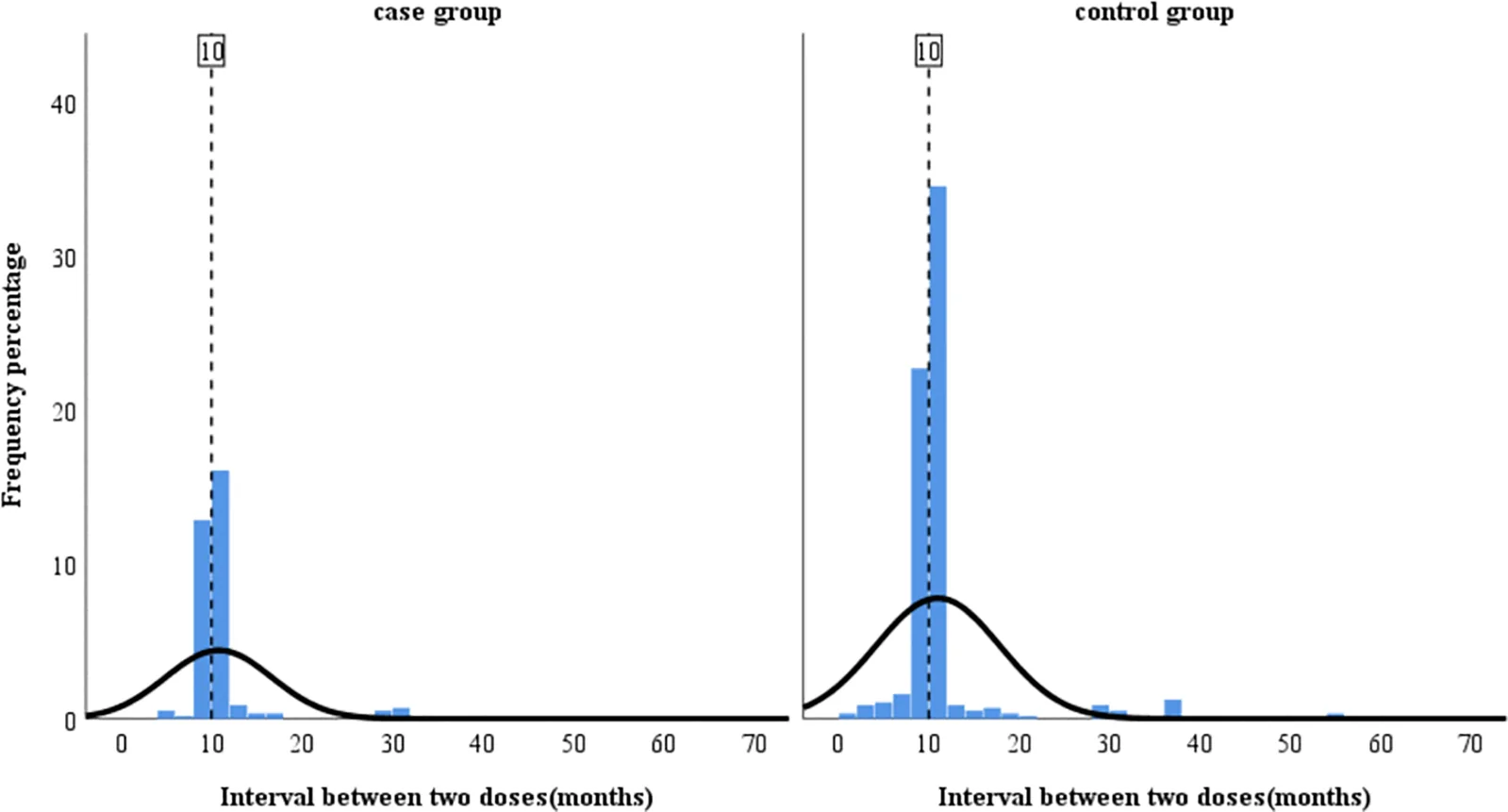
Distribution of interval between two doses (months). Note: The left panel shows the case group and the right panel shows the control group. The dashed line indicates the cutoff (10 months). Both groups showed a concentration around 10 months, supporting the use of 10 months as the cutoff.

Based on the data distribution patterns and commonly used standards in comparable domestic studies, we determined 18 months, 5 years, and 10 months as the cutoff values for grouping, and the grouping scheme was considered reasonable.

## Discussion

Based on real-world data from a 1:2 matched case-control study design, this study included 1405 cases and 2810 controls. The case and control groups were well-balanced regarding key demographic characteristics such as sex, stage of schooling, residence, and birth period, demonstrating good comparability between groups and providing a reliable data foundation for the objective evaluation of MuCV vaccine effectiveness. In terms of sex distribution, males accounted for 62.9% of the case group, compared to 37.1% for females. This male predominance is consistent with the known epidemiological pattern of mumps, which typically shows higher incidence in males than in females [27]. The sex ratio in the general population of children under 15 years of age in Taizhou City was approximately 1.1:1 (male:female), suggesting that the observed male proportion in this study was higher than the population background level, further supporting that males may have higher susceptibility to mumps infection.

The study showed that receiving MuCV is a key and effective protective factor against mumps. Children who received at least one dose of MuCV had a VE of 75.7% compared to unvaccinated children, a result consistent with both domestic and international research [28–30], further confirming the good immunogenicity and protective efficacy of MuCV. It is noteworthy that the overall MuCV vaccination coverage among children in the study area was extremely high, with unvaccinated individuals accounting for only 0.5% of the control group. Under such high vaccine coverage, families who were vaccinated and more engaged with health services may have been more likely to participate in the study, potentially leading to an overestimation of VE. Across different stages of schooling, the protective effectiveness of MuCV exhibited a significant trend of increasing with age. However, this finding should be interpreted with caution. In the context of high vaccination coverage, the extremely small number of unvaccinated children in some age groups led to unstable *OR* estimates and wide confidence intervals in certain subgroups, posing a risk of sparse data bias. Traditional logistic regression may yield unreliable estimates or even overestimate VE values [31,32]. After performing Firth penalized logistic regression sensitivity analyses, the corrected VE estimates for each schooling stage were generally consistent with the main analysis, and the conclusions remained unchanged. Therefore, the age-related trend in VE should not be directly interpreted as a true enhancement of protection with increasing age; this result should be considered an exploratory finding rather than a confirmatory conclusion. Regarding the lack of significant protective effect observed in infants and young children, the potential reasons are analyzed as follows. On one hand, the immune system of infants and young children is not yet fully developed, with significantly lower immune cell counts and cytokine activity compared to older children. Following MuCV vaccination, their ability to mount a specific immune response is relatively weaker, and antibody levels and persistence may be insufficient, thereby limiting the full manifestation of vaccine protection [33]. On the other hand, our data show that the two-dose vaccination coverage among infants and young children has reached 61.0%, the highest among all age groups, indicating that “incomplete vaccination” is not the primary reason for the suboptimal protection in this age group. However, infants and young children are in the initial stage of group living, with high population density and poor hygiene behavior compliance, resulting in significantly higher virus exposure risk than other age groups [27]. Even when vaccinated, high exposure burden may attenuate the real-world effectiveness of the vaccine. Furthermore, differences in VE across stages of schooling may also reflect the combined effects of exposure intensity and immune status across populations. The VE among kindergarten children was 55.2%. Although differing in specific value from the VE of 16.88% reported in a study conducted in Hefei City by Tingting Shi et al. [1], both results indicate a relatively low VE in this age group. This finding aligns with the recent epidemiological trend of mumps in China: between 2020 and 2023, the age group with the highest mumps incidence shifted forward, with the proportion of cases in the 0 ~ 4 age group increasing while the proportion in the 10 ~ 14 age group decreased. The age group with the peak incidence shifted from 7 years to 4 years [17], further confirming that kindergarten children are a high-risk group for mumps infection [34]. These changes highlight that infants, young children, and kindergarten children are becoming the vulnerable and key populations for current mumps prevention and control. In this study, VE significantly increased to 86.7% and 92.9% among primary and secondary school children, respectively. A birth cohort study based on mumps incidence data in Beijing from 2005 to 2016 showed that the protective effectiveness of MuCV exhibited an upward trend with birth cohorts [35], which is consistent with our findings. Furthermore, a Meta-analysis evaluating MuV antibody levels and immune effectiveness in the Chinese healthy population indicated that the positive rate of mumps virus IgG antibodies increased with age and the number of vaccine doses received [36]. Based on the findings of this study and the local epidemiological characteristics of mumps, it is necessary to prioritize catch-up vaccination for infants, young children, and kindergarten children, improve the timeliness and completion rate of vaccination, and close the immunity gaps in younger populations. Since the present study did not find a significant incremental protective effect of the second dose compared with the first dose, future studies with larger sample sizes are needed for further verification.

A noteworthy finding is the markedly different protective effectiveness of MuCV observed between the two periods of immunization schedule implementation. Previous research has shown statistically significant differences in the average annual mumps incidence between different immunization periods [37]. During the period of the single-dose immunization schedule, MuCV demonstrated a significant protective effect (VE = 80.4%), which may stem from a substantial difference in the unvaccinated rates between the case and control groups. However, during the period of the two-dose immunization schedule, vaccination did not show a statistically significant protective effect. In-depth analysis revealed highly similar vaccination distributions between the two groups during this period, with nearly identical proportions of unvaccinated individuals and those who received 1 or 2 doses. This result likely does not indicate vaccine failure, but rather reflects that, after the two-dose vaccination has become highly prevalent, there was no significant difference in vaccination status between cases and controls, making it difficult for the traditional case-control study design to detect an incremental protective effect. In addition, the relatively small sample size during this period (only 163 cases in the case group) may have affected statistical power. In the context of high vaccination coverage, sparse data (e.g., very few unvaccinated individuals) and sample size limitations may lead to unstable estimates from traditional logistic regression, which to some extent constrains the accurate assessment of the incremental protective effect of the two-dose strategy. Therefore, the results of this study should not be interpreted as evidence that the two-dose schedule is ineffective, but rather as an indication that more refined study designs (such as cohort studies) are needed to evaluate vaccine effectiveness under high coverage conditions. However, the influence of factors such as viral epidemic characteristics and prevention and control measures cannot be ruled out. Studies from multiple regions in China have shown that non-pharmaceutical interventions implemented during the COVID-19 pandemic from 2020 to 2022—including mask-wearing, hand hygiene, and reduced population movement—were associated with reduced mumps incidence [27]. After 2023, China’s COVID-19 prevention and control policies were adjusted. Mask-wearing requirements in public places were gradually lifted, schools and childcare facilities resumed normal operation, and children’s extracurricular activities and social gatherings increased markedly. Close contact opportunities among children rebounded significantly compared with the pandemic period, which may have facilitated exposure of susceptible children to the mumps virus. However, as we did not systematically collect data on the intensity and timing of non-pharmaceutical intervention implementation, we could not quantitatively assess their impact on VE estimates.

Using unvaccinated individuals as the reference, we categorized vaccinated participants into two groups according to age at first dose: < 18 months and ≥18 months. The results showed that the P values for the two groups were 0.456 and 0.460, respectively, with no statistical significance, which is consistent with previous findings [38]. Because only 14 unvaccinated controls were available, the *OR* and VE estimates were extremely unstable, indicating complete separation and sparse data bias, making it impossible to reliably estimate the protective effect. Therefore, the impact of age at first dose on vaccine effectiveness remains uncertain. A study on the immune response to the F-genotype mumps attenuated live vaccine in infants of different ages showed no significant difference in immune response between younger and older infants after vaccination [39]. However, a study based on mumps incidence data in Jiangsu Province from 2004 to 2019 indicated that the seroconversion rate (16.0%) for the currently dominant epidemic strain (F-genotype wild strain) was lower for vaccination starting at 8 months compared to starting at 18 months (65.1%) [40]. However, vaccine effectiveness is influenced by multiple factors, including population exposure levels, circulating strain characteristics, and completeness of the immunization schedule. Nevertheless, under China’s current immunization schedule, initiating MMR vaccination at 8 months of age appears to be effective, and there is no need to delay vaccination due to concerns about insufficient immune responses in infants and young children. This has important public health implications for controlling early transmission of mumps in children and reducing the risk of severe disease and complications. It should be noted, however, that age at first dose is highly correlated with the immunization schedule period—children who received their first dose at <18 months were predominantly from the two-dose schedule period, whereas those who received their first dose at ≥18 months were mainly from the single-dose schedule period. These two factors are closely intertwined, making it difficult to completely disentangle the independent effects of age at first dose from those of the immunization schedule period on VE. Therefore, the impact of age at first dose on the protective effect remains uncertain, and this conclusion should be interpreted with caution in this context. Sensitivity analyses also showed no significant linear association between age at first dose and the risk of mumps, and the Firth penalized logistic regression results were consistent with the main analysis. The robustness of this conclusion should be understood in light of the aforementioned confounding scenario.

Regarding the selection of the cutoff for time since last dose, the choice of 5 years was primarily based on the data distribution characteristics. As shown in the distribution plots, using 5 years as the cutoff resulted in more balanced sample sizes between the two groups and more stable OR estimates. Among individuals who had received at least one dose of vaccine, this study did not detect a significant effect of time since last dose (≤5 years vs. > 5 years) on vaccine effectiveness.A comprehensive analysis of French data by Sabine Vygen et al. showed that the risk of mumps increased by 7% for each additional year since the last MMR dose [41]. Surveillance results of mumps antibody levels in a healthy population in Sanmenxia City, Henan Province, from 2019 to 2023 showed the lowest mumps IgG antibody positive rate (70.24%) in the group with 5 to <10 years since the last dose, and the highest (85.63%) in the group with 2 to <3 years [42]. To further explore long-term waning immunity, we stratified time since last dose among one-dose recipients into four categories: ≤ 2 years, 2 ~ 5 years, 5 ~ 10 years, and >10 years. The results showed that the > 10 years group reached statistical significance, suggesting that detectable waning of protection following a single dose of MuCV may only become apparent after 10 years or more. When time since last dose was analyzed as a continuous variable or using Firth penalized logistic regression, no significant association with the risk of mumps was observed. Taken together, these results suggest that no significant effect of time since last dose on vaccine effectiveness was detected within 10 years in this study; however, the data indicate a trend toward waning immunity beyond 10 years. Given the limited sample size (particularly the small number of participants in the > 5 years subgroup), these findings should be considered exploratory and warrant further investigation.

Among children who completed the two-dose vaccination series, this study did not find a significant impact of the interval between the two doses (≤10 months vs. > 10 months) on vaccine effectiveness. This aligns with findings from a French study [41], but differs from several domestic studies reporting a significant association between the two-dose interval and the risk of mumps [23,38,43]. This discrepancy may be related to different definitions of interval grouping across studies. This study, based on vaccination timing data from mumps cases under 15 years of age occurring between 2021 and 2025, divided the interval into two groups: ≤ 10 months and >10 months. In contrast, Li Xuemei et al. [38] used intervals of ≤12 months and 13–36 months, Wang Haibo et al. [23] used 4 years as the cutoff, and in the study by Zhang Chao et al. [43], the interval from vaccination to disease onset was concentrated between 2 and 6 years. Different grouping definitions and interval spans may lead to variations in study results. Regarding the two-dose MuCV interval, the optimal age for the second dose varies significantly among countries [44]. A 10-year follow-up study on the long-term immunogenicity of MuCV in healthy young children across 10 countries showed that the second dose of MuCV does provide a temporary boost in antibody levels, and this effect was independent of the timing of the second dose (whether administered 42 days to 9 ~ 10 years after the first dose) [45]. The results of this study should be interpreted cautiously: within the observed range of two-dose intervals, appropriate adjustments to the interval do not significantly affect vaccine effectiveness. However, this does not preclude the possibility that an extremely long interval might lead to reduced protection. Sensitivity analyses also revealed no significant linear association between the interval between two doses and vaccine effectiveness. This finding was further supported by the Firth penalized logistic regression analysis, suggesting that, within the observed range of two-dose intervals, appropriate adjustment of the interval does not significantly affect vaccine effectiveness.

In summary, it is recommended that priority be given to vaccination management for infants, young children, and kindergarten children at this stage, with efforts to improve vaccination timeliness and full-course coverage, and to reduce immunity gaps. Future efforts could include expanding sample sizes, conducting multicenter long-term cohort studies and serological surveillance to explore the feasibility and cost-effectiveness of adjusting immunization strategies for younger children. Concurrently, immunization planning should be continuously optimized in light of changes in viral epidemic characteristics, providing more robust epidemiological and immunological support for the scientific prevention and control of mumps in children.

## Conclusion

Based on a 1:2 matched case-control study using real-world data from children under 15 years in Taizhou City, this study found that MuCV vaccination provides significant overall protection against mumps in children. Stratified analysis suggested that vaccine effectiveness may increase with age; however, it should be noted that the extremely small number of unvaccinated individuals in some age groups under the high-coverage background may affect statistical estimation, and this finding should be considered exploratory. Regarding different immunization schedule implementation periods, a significant protective effect was observed during the single-dose period (VE = 80.4%), whereas no statistically significant protective effect was detected during the two-dose period. This result should not be interpreted as evidence that the two-dose schedule is ineffective; rather, it may be related to the highly similar vaccination distributions between cases and controls and the limited sample size. Using unvaccinated individuals as the reference, the *P* values for both the < 18 months and ≥18 months groups were not statistically significant. However, given the extremely small number of unvaccinated controls, *OR* and VE could not be reliably estimated, and the impact of age at first dose on vaccine effectiveness remains uncertain. The interval between two doses did not show a significant effect on vaccine effectiveness. Regarding time since last dose, dichotomization at 5 years (≤5 years vs. > 5 years) did not reveal statistical significance. However, in one-dose recipients, the > 10 years group reached statistical significance, suggesting that detectable waning of protection following a single dose may only become apparent after 10 years. Given the limited sample size, this finding should be considered exploratory. Moreover, due to the relatively small sample sizes in these subgroups, the conclusions should be cautiously interpreted as “this study failed to demonstrate the effects of these factors” rather than concluding that they have “no effect.”

Based on the findings of this study, mumps prevention and control efforts in this region should continue to consolidate and improve full-course vaccination coverage, with a particular focus on strengthening vaccination management and catch-up vaccination for infants, young children, and kindergarten children. The lack of detectable incremental effect during the two-dose period in this study likely reflects high baseline coverage rather than vaccine failure, and therefore should not be used as evidence against the two-dose schedule. It is recommended to further integrate routine epidemic surveillance and serological antibody level monitoring to dynamically assess vaccine effectiveness and optimize immunization strategies, thereby continuously strengthening population immunity and effectively reducing the incidence of mumps.

## Strengths and limitations

This study employed a 1:2 matched case-control design, including 1405 cases and 2810 controls. The adequate sample size ensured high statistical power. The results likely reflect the real-world protective effectiveness of MuCV well, offering strong generalizability and public health significance. Multidimensional stratified analyses were conducted using conditional logistic regression models. While controlling for potential confounders, the study systematically explored the impact of multiple dimensions—including the number of doses, stage of schooling, stage of immunization schedule implementation, age at first dose, time since the last dose, and interval between two doses—on VE. This helps address gaps in previous research and provides epidemiological evidence for optimizing MuCV vaccination strategies for children.

Despite careful consideration in the design and analysis, this study has certain limitations. First, as a retrospective matched case-control study, it cannot completely avoid selection bias and information bias. Case data were sourced from the China Disease Prevention and Control Information System, and differences in diagnostic criteria and reporting quality across healthcare facilities at different levels may introduce selection bias in case inclusion. Vaccination information was obtained from immunization certificates and the immunization program information system. Although the data completeness is relatively high, critical information such as vaccine lot number and manufacturer was unavailable, making it difficult to conduct further in-depth analyses on vaccine brand or specific lots. Additionally, among the 1,498 cases initially meeting the inclusion criteria, 93 were lost to follow-up due to refusal to participate, resulting in a loss-to-follow-up rate of 6.2%. If systematic differences exist between the lost-to-follow-up cases and the included cases, this could potentially influence the estimation of VE. We also noted that the measured MuCV vaccination coverage among enrolled controls was 99.5%; however, given the 6.2% non-response rate in the case group, this coverage may not fully represent the true population vaccination level, and non-response bias may have a potential impact on the VE estimates. Second, while major demographic confounders were controlled through matching, residual confounding from factors such as children’s nutritional status, household exposure levels, and the intensity of outbreaks in childcare or school settings cannot be excluded. Third, the sample sizes for several subgroup analyses were limited (e.g., zero unvaccinated controls in the kindergarten group; and only 163 cases during the two-dose period), resulting in insufficient statistical power to detect differences in small effect sizes. Therefore, some subgroup findings should be considered exploratory rather than confirmatory. In addition, the absence of unvaccinated individuals in certain subgroups (e.g., kindergarten and primary school control groups) posed a risk of sparse data bias [44]. To address this, we conducted sensitivity analyses using Firth penalized logistic regression, and the results showed that the effect estimates were generally consistent with the main analysis, suggesting that sparse data bias had limited impact on the core conclusions. Although the categorization of continuous variables was based on data distribution characteristics and commonly used standards in comparable domestic studies, and the continuous variable sensitivity analyses were consistent with the subgroup analyses, this approach may still have masked some non-linear relationships. In the analysis of time since last dose, the sample size in the > 10 years group was limited (358 cases and 713 controls among one-dose recipients), and the *OR* estimates had wide confidence intervals, making it difficult to fully evaluate the long-term waning of vaccine-induced immunity. Fourth, the proportion of laboratory-confirmed cases in this study was relatively low. Future studies should strengthen laboratory testing capacity and increase the proportion of laboratory-confirmed cases to reduce the interference of clinical misdiagnosis on VE estimation. In addition, the study period coincided with the COVID-19 pandemic, during which non-pharmaceutical interventions such as mask-wearing, social distancing, and temporary closure of schools and childcare facilities were implemented. These measures may have reduced the transmission of mumps to some extent and potentially influenced VE estimates during the two-dose implementation period. Since we did not systematically collect data on the intensity and timing of these measures, we could not precisely assess their impact on the study results, and this should be taken into consideration when interpreting the findings.
